# Supplementary material for: Association between oral microbiome and seven types of cancers in East Asian population: a two-sample Mendelian randomization analysis
Source: Front Mol Biosci. 2023 Nov 21;10:1327893. doi: 10.3389/fmolb.2023.1327893 (PMC10702768; doi:10.3389/fmolb.2023.1327893)
Supplement: Supplementary file 1 [file DataSheet1.ZIP › Supplementary Materials/MR plots of saliva/saliva═╝/Prostate cancer/pheno.2608_to_prostate cancer_forest.pdf]

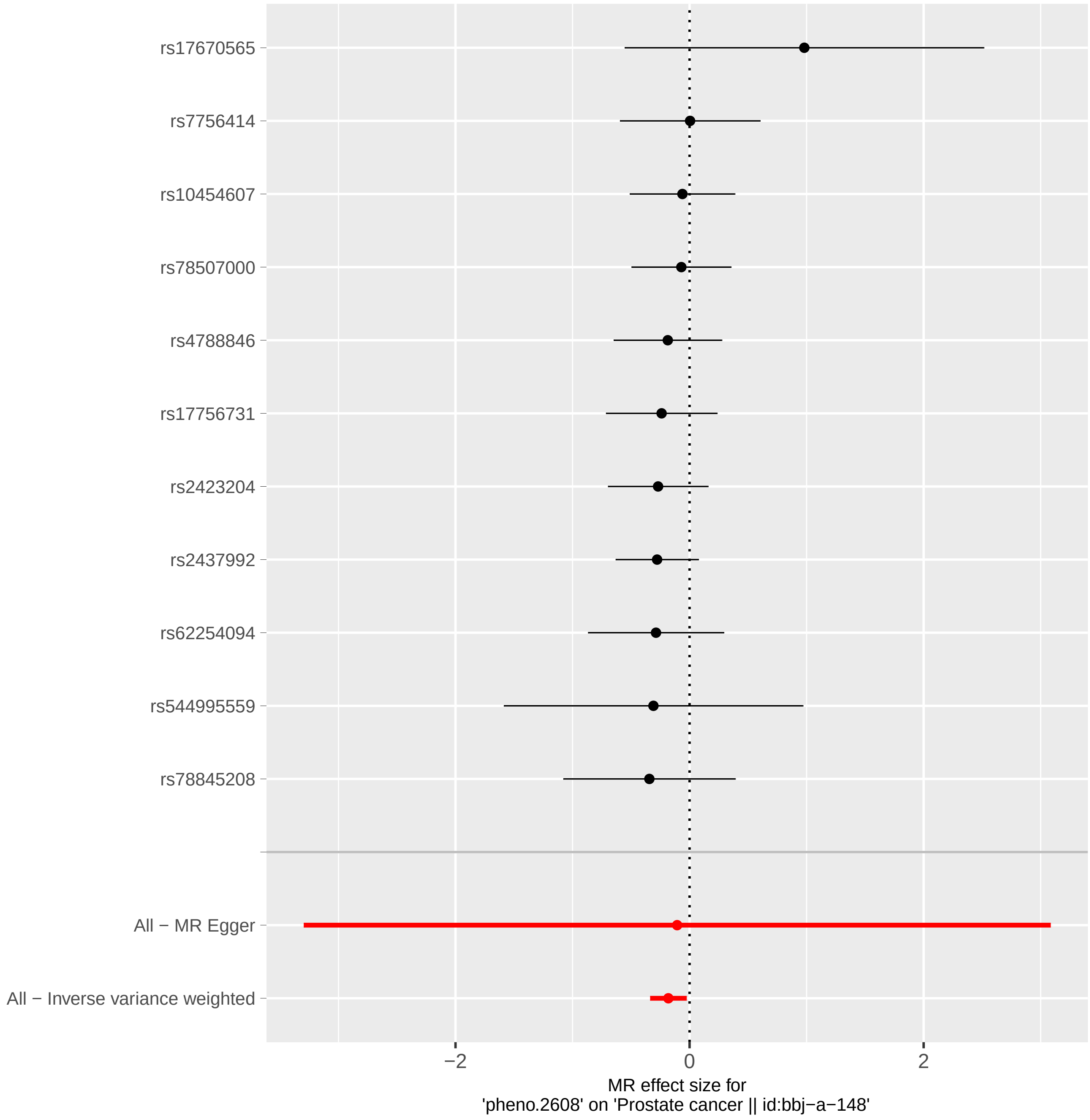
rs17670565
rs7756414
rs10454607
rs78507000
rs4788846
rs17756731
rs2423204
rs2437992
rs62254094
rs544995559
rs78845208
All − MR Egger
All − Inverse variance weighted
−2
0
2
MR effect size for
'pheno.2608' on 'Prostate cancer || id:bbj−a−148'
